# Dealing with the Problem of Monotone Likelihood in the Inflation of Estimated Effects in Clinical Studies. Comment on Hasegawa et al. Impact of Blood Type O on Mortality of Sepsis Patients: A Multicenter Retrospective Observational Study. *Diagnostics* 2020, *10*, 826

**DOI:** 10.3390/diagnostics12102295

**Published:** 2022-09-23

**Authors:** I-Shiang Tzeng

**Affiliations:** 1 Department of Research, Taipei Tzu Chi Hospital, Buddhist Tzu Chi Medical Foundation, New Taipei City 23142, Taiwan; istzeng@gmail.com; 2 Department of Statistic, National Taipei University, Taipei 10478, Taiwan; 3 Institute of Education, Tzu Chi University, Hualien 97004, Taiwan

I read the press article from the Fujita Health University School of Medicine titled “Impact of Blood Type O on Mortality of Sepsis Patients: A Multicenter Retrospective Observational Study”. It was based on an article by Hasegawa et al. [1]. This study aimed to explore the association between the risk of mortality and blood type for sepsis patients based on a retrospective cohort study. The authors found that sepsis patients with blood type O are associated with a significantly higher risk of 90-day mortality from septic shock. Indeed, this research has provided important contributions to the field; however, there are some biostatistics issues that should be clarified.

Firstly, the authors compared the risk of 90-day mortality in patients with septic shock between blood type groups and found that the adjusted odds ratio (OR) of mortality increased 3.26 times in patients with blood type O compared to that in the blood type non-O reference group (blood type O patients with sepsis shock (death number/total number = 21/40), blood type non-O patients with sepsis shock (death number/total number =21/76); adjusted OR = 3.26; 95% confidence interval (CI: 1.34–7.9)). The massive effect estimation that may result from specificity in the data set, called “monotonic likelihood” [2,3], is of concern to scientists. The true OR cannot be confirmed if the CI of the OR is too wide. We suggest that the researchers reconstruct the interval estimation based on profile penalized log likelihood (PPL) owing to the wide CI with a massive effect estimate [3].

In addition to the longitudinal cohort study, a similar phenomenon was observed for the odds ratio (OR) in a cross-sectional cohort study. As a demonstration, a lung cancer data set [4] was analyzed with the package logistic [5], which applies the Firth regression in the software R (version 4.0.0, Vienna, Austria). A total of 649 male patients with cancer were included (cases), 647 of whom were reported to be smokers. An equal number of participants (649 men without cancer) were recruited as the control group, 622 of whom were reported to be smokers. The OR of lung cancer in smokers compared with that of non-smokers can be calculated as (647 × 27)/(2 × 622) = 14.04—that is, the odds of contracting lung cancer in smokers was estimated to be 14 times the odds of contracting lung cancer in non-smokers. We would like to determine the reliability of this estimate based on the width of the 95% CI. The 95% CI for this OR is between 3.33 and 59.3. After modification based on PPL, the OR and 95% CI of lung cancer for smokers compared with that of non-smokers decreased (modified OR = 11.44; 95% CI: 3.74–56.42). Figure 1 shows that the range of the 95% CI of smoking status, which was estimated based on a PPL, is shorter than that of the 95% CI before modification.

As shown in Table 3 of the study conducted by Hasegawa et al. [1], only 21 cases were present in blood type O of patients with septic shock, which means that their data were subject to sparse events, which may have increased the probability of monotone likelihood. Several methods have been proposed to solve the problem of monotonic likelihood through data penalization, and augmentation has been suggested as an efficient method [2]. After adjusting for age, sex, and Sequential (Sepsis-related) Organ Failure Assessment score in Table 4 [1], we found that the effect estimate was eliminated and the CI was significantly increased (adjusted OR = 3.26; 95% CI: 1.34–7.9 in contrast to crude OR = (21 × 76)/(21 × 40) = 1.9, 95% CI: 0.93–3.89 calculated by a website was available at https://www.medcalc.org/calc/odds_ratio.php, accessed on 1 June 2022). We speculate that this problem is not limited to univariate models alone [2]. When considering multiple regressions, the sample size, event number, dimension of association with the result, size of the balance, a linear combination of variables [6,7], and count of binary covariates affect the generality of monotonic likelihood. This is a comment article aiming to evaluate whether the estimate of the 90-day mortality risk of blood type O (i.e., inherent risk factor) patients with septic shock may have had a bias. As this was a retrospective observational study [1] with several potential sources of error in the measurement of the sepsis event and independent variables, the point estimates must be seen as approximate and certainly the accuracy would have been increased by the PPL modification suggested by the author of the letter. However, we also must not overemphasize the magnitude of those non-sampling errors. Therefore, we commented on a study [1] that may be a potentially useful note for researchers involved the problem of the monotonic likelihood of the univariate and multivariate models in clinical studies.

## Figures and Tables

**Figure 1:**
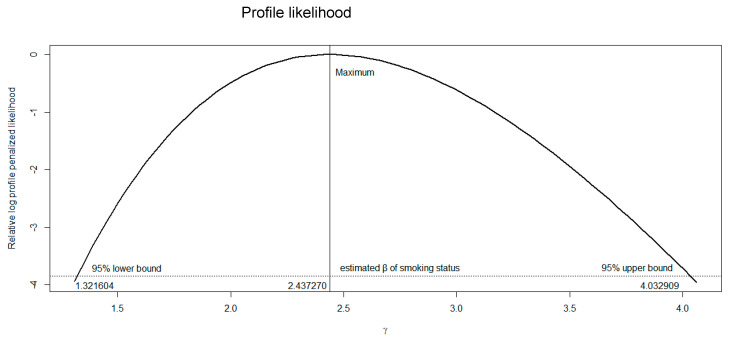
Plot of the profile penalized log-likelihood function for variable smoking status.

## Data Availability

Data was available at https://www.mdpi.com/2075-4418/10/10/826.
